# Cardio-vascular tissue processing

**DOI:** 10.1186/1749-8090-8-S1-O104

**Published:** 2013-09-11

**Authors:** Ramadan Jashari

**Affiliations:** 1 European Homograft Bank (EHB) International Association, Brussels, Belgium

## 

The Cardio-vascular Tissues aimed for clinical use are submitted to the European Regulation (Directives 2004/23EC, 2006/17EC and 2006/86EC) to assure high level of tissue quality and security. The European Directive was transposed into the Member state legislation and Standards, specific to their needs and possibilities.

The Cardio-vascular tissues are recovered in the operating theatre or another area which has a controlled environment conform to the GMP regulation. The aseptic infrastructure is mandatory for all steps of the tissue recovery, transport and processing.

Processing of cardio-vascular tissues has to be carried out in a class A laminar flow conditions, background class C, with fully controlled environment for particles and aerobe, anaerobe germs and fungi. The temperature and the air pressure need to be adapted to the GMP regulation, assuring the pressure-cascade with a higher level at processing area and lower pressure around. The substances that come in contact with tissues (Hanks, dimethyl-sulfoxide, Antibiotics) that are mandatory for assuring the quality of tissues, may not provoke any deterioration of the tissue, assuring that the cells and matrix remain intact at the end of processing. Morphology, measurements, functional test and cryopreservation and storage have to be strictly respected and notified for the implanting surgeon.

Regular environmental control is mandatory, in order to guarantee the “sterile conditions” in zone A, where the tissue is processed. The critical procedures have to be mentioned in the Quality Management System (QMS) and validated conform the type of the tissue. Any kind of non-conformity during the tissue processing must be notified and corrected.

The Tissue Establishment needs appropriate staff and equipment. The QMS has to be established and contain following elements: Quality Manager and a replacing person, a manual of quality which describes all the procedures and the way of managing the quality system, and the SOP (Standard Operating Procedures) which describe any kind of procedures regarding tissue processing. Of course, the appropriate equipment and material is needed. On top of all, a Medical Doctor has the entire responsibility of the Tissue Establishment.

Documenting of all steps of tissue processing from the recovery to the implantation of tissues is mandatory.

